# Health-Related Internet Use Among Outpatients Undergoing Cancer Treatment During the COVID-19 Pandemic: Cross-Sectional Survey Study

**DOI:** 10.2196/72614

**Published:** 2025-08-14

**Authors:** Nicole Erickson, Daniel Nasseh, Sarah Seynstahl, Nicole Jost, Rachel Wuerstlein, Jozefina Casuscelli, Sebastian Theurich, Volker Heinemann, Theres Fey

**Affiliations:** 1 Comprehensive Cancer Center, Ludwig Maximilian University Hospital Munich, Pettenkoferstrße 8a, Munich, 80336, Germany, 49 89 4400 75218; 2 Department of Obstetrics and Gynecology, Ludwig Maximilian University Hospital Munich, Munich, Germany; 3 Department of Urology, Ludwig Maximilian University Hospital Munich, Munich, Germany; 4 Department of Medicine III, Ludwig Maximilian University Hospital Munich, Munich, Germany; 5 Bavarian Cancer Research Center, Munich, Germany

**Keywords:** eHealth, mobile health, health-related internet use, health literacy, patients with cancer, COVID-19, oncology

## Abstract

**Background:**

Health care professionals and patients frequently use the internet to access medical information. However, health-related mobile apps have yet to become fully integrated into routine clinical practice. Although the demand for eHealth apps has grown only modestly, studies suggest that such tools hold significant potential to enhance medical care and improve the quality of life, particularly for patients with cancer. To successfully implement these technologies in everyday practice, it is essential to understand the specific needs and preferences of the target population.

**Objective:**

The aim of this study was to assess internet and eHealth use among patients with cancer receiving chemotherapy at an outpatient ward and to evaluate how the COVID-19 pandemic influenced internet and eHealth app use.

**Methods:**

Between May 2021 and September 2021, a total of 303 patients receiving outpatient care at the hemato-oncology and gynecology departments of a Comprehensive Cancer Center were surveyed using a 21-item paper-based questionnaire, adapted from a validated information and communication technology–use survey. The questionnaire provided patient-reported information related to general internet abilities and use rates including health-related apps and changes during the COVID-19 pandemic. Data analysis was conducted using descriptive statistics, chi-square tests, Mann-Whitney *U* tests, and Spearman correlations.

**Results:**

In total, 98.7% (299/303) of participants reported regular internet use, 72.6% (217/299) reported using the internet to search for health-related information, and 79.1% (235/297) expressed readiness to communicate digitally with health care providers. Decreasing age and higher internet literacy correlated with a more frequent use of eHealth apps (*P*<.001). A total of 24.7% (68/275) reported increased internet use during the pandemic.

**Conclusions:**

The majority of patients were regular internet users and expressed an openness to eHealth apps. Factors such as internet literacy and average age are important to consider when implementing new eHealth apps in a clinical setting. Despite the positive influence of the pandemic on internet use, there remains a gap between self-reported readiness and real use of eHealth apps.

## Introduction

The internet and its wide scope of application possibilities is indispensable in our everyday life. In Germany, approximately 92% of the population uses the internet daily, and the number of nonusers decreases steadily every year [1,2]. While internet use is more common among the younger age groups, data show that already over 90% of people younger than 65 years of age use the internet daily [2]. Internet use among the older generation is concurrently steadily increasing. According to recent data, 27.7% of the internet users are ≥60 years of age [3,4].

At the same time, the importance of digital technologies in health care is rising. This trend is captured under the term “eHealth,” defined as the cost-effective and secure use of information and communication technologies in support of health and health-related fields [5,6]. In the medical context, the internet is used particularly frequently by medical professionals and patients to search for information [7-9]. However, the adoption of more advanced digital health tools—particularly mobile health apps—remains limited. These apps extend beyond mere information retrieval and offer functionalities such as patient-provider communication, symptom monitoring, therapy management, and the collection of patient-reported outcomes [10]. Practical examples of these apps include the Corona-Tracing-App [11], the digital COVID-19 European Union–vaccination certificate [12], or cancer-specific solutions such as CANKADO [13-15]. Despite their potential, such tools have not yet been widely integrated into routine clinical care [16]. Nevertheless, van Eenbergen et al [17] documented that the demand for eHealth apps among patients has increased to a very small extent in the years between 2005 and 2017 [9,17].

Among patients with cancer, internet use is often driven by the desire to understand their disease and treatment options and to connect with other patients or peer support groups [8,18]. While approximately three-quarters of patients with cancer turn to the internet for health-related information, only about half of these users are considered autonomous—able to navigate the internet independently without external assistance [18]. Despite this widespread use for informational purposes, only a small fraction of patients actively engage with more advanced eHealth apps beyond basic web-based searches [7,18-21].

Nevertheless, emerging evidence indicates that the use of eHealth apps has the potential to improve medical care and increase quality of life among patients with cancer. Some studies even demonstrate a link between eHealth app use and an improvement in overall and progression-free survival [22,23]. However, before introducing and using such tools in clinical practice, it is important to understand the target group. Therefore, the aim of this cross-sectional single-point cohort study was to determine if patients with cancer receiving outpatient treatment within our Comprehensive Care Center have access to, and are open to, the use of digital health apps.

## Methods

### Recruitment

This single-point cohort study was conducted at the outpatient hemato-oncology and gynecology departments of the Comprehensive Cancer Center at LMU Klinikum (Ludwig-Maximilians-University Hospital) in Munich, Germany. LMU Klinikum is one of the largest university hospitals in Europe, serving a diverse patient population from both urban and rural regions across Bavaria.

Between May 1, 2021, and September 30, 2021, patients receiving outpatient care in the aforementioned departments were invited to participate. Study personnel screened patients daily to assess eligibility. All patients aged 18 years or older were eligible for inclusion. Individuals with significant language barriers or functional impairments, as assessed using the Eastern Cooperative Oncology Group performance status, were excluded.

Eligible patients were approached in person during their scheduled visits and received verbal information about the purpose of the study. The survey was paper-based, completed on-site, and required approximately 5 to 10 minutes to complete. Assistance was offered if needed.

Participation was entirely voluntary. Due to the anonymous nature of the data collection, no personal or demographic information was recorded for nonparticipants. As a result, no formal comparison between participants and nonparticipants could be conducted. However, we acknowledge the potential for selection bias—particularly a tendency toward participation among individuals with higher digital literacy or a greater interest in digital health. This may limit the generalizability of the findings to populations with lower levels of internet access or digital affinity.

### Questionnaires

The 21-item questionnaire was a modified version of the extended “use of information and communication technologies questionnaire (ICT) developed and validated by Seifert et al [24] for people aged 65 years and older in Switzerland in 2015” (Multimedia Appendix 1).

The first 3 of the 21 items collected basic demographic data including gender, year of birth, and diagnosis. The second part, the core of the questionnaire, consisted of 9 items relating to aspects of the patient’s internet use. Analogous to Seifert et al [24], we collected data regarding which internet-enabled devices they own in the household (ie, computer and mobile), which one of these devices they most frequently use, and how often they use the internet. Patients who did not use the internet were asked for reasons. Furthermore, we gathered information regarding the age, the operating system, and the software of the mobile device. The remaining items referred to the patient’s ability to use the internet and install apps.

The third part of the questionnaire consisted of 6 items related specifically to eHealth apps. We asked the patients to specify which eHealth apps they already use and which type of eHealth apps they would be willing to try to use.

The final 3 items focused on internet use during the COVID-19 pandemic. The first 2 items referred to the use of the “Corona-Warn-App,” which was developed and released by the German government in order to simplify and expand contact tracing [25]. The last item used self-reported data to elicit information regarding how the pandemic has changed the frequency of internet use and if they have begun using eHealth apps during the pandemic. Patients were defined as “completers” when 90% of the items on the questionnaire were filled out (ie,≤2 items of 21 items were missing). All data are therefore presented based on completers only.

In future studies, the inclusion of open-ended questions—such as patient suggestions for desired features or concerns about digital health tools—may provide valuable qualitative insights and complement the quantitative findings.

### Statistical Analysis

All data analysis was conducted with SPSS (version 26; IBM Corp) for Windows. Demographics and recruitment data were analyzed using descriptive statistics. Descriptive statistics were also used for analyzing internet user patterns of patients. The 2-tailed *t* test and Mann-Whitney *U* test were used to analyze group differences for numeric variables (ie, number of used apps). The chi-square test of independence and contingency analyses were used to analyze categorical variables. The Spearman correlation analysis was chosen to explore associations between ordinal variables (eg, perceived digital confidence and willingness to use eHealth) and continuous variables (eg, age), as many of the relevant variables were nonnormally distributed or measured on an ordinal scale. After analyzing the complete dataset, the data were then reanalyzed for differences between the 2 cohorts of patient groups from the 2 different departments (hemato-oncology and gynecology) using Mann-Whitney *U* tests and chi-square tests of independence. All tests were 2-sided, and the significance level was set to .05.

### Ethical Considerations

The Medical Ethics Committee of the University of Munich determined that no formal ethics approval was required for this study, as the data were collected entirely anonymously and no personal identifiers were retained (project: KB 20/015). This decision is in accordance with institutional policies for research involving fully anonymized and nonreversibly deidentified data. Participation in the study was entirely voluntary. Patients were informed verbally about the purpose and content of the questionnaire during their outpatient visit, and the completion of the anonymous questionnaire was considered as implied informed consent. All data were collected and processed in a way that ensured full anonymity; no personally identifying information was recorded, and responses were nonreversibly anonymized prior to analysis. No compensation was provided to participants for their participation in the study.

## Results

### Overview

**Figure 1.**
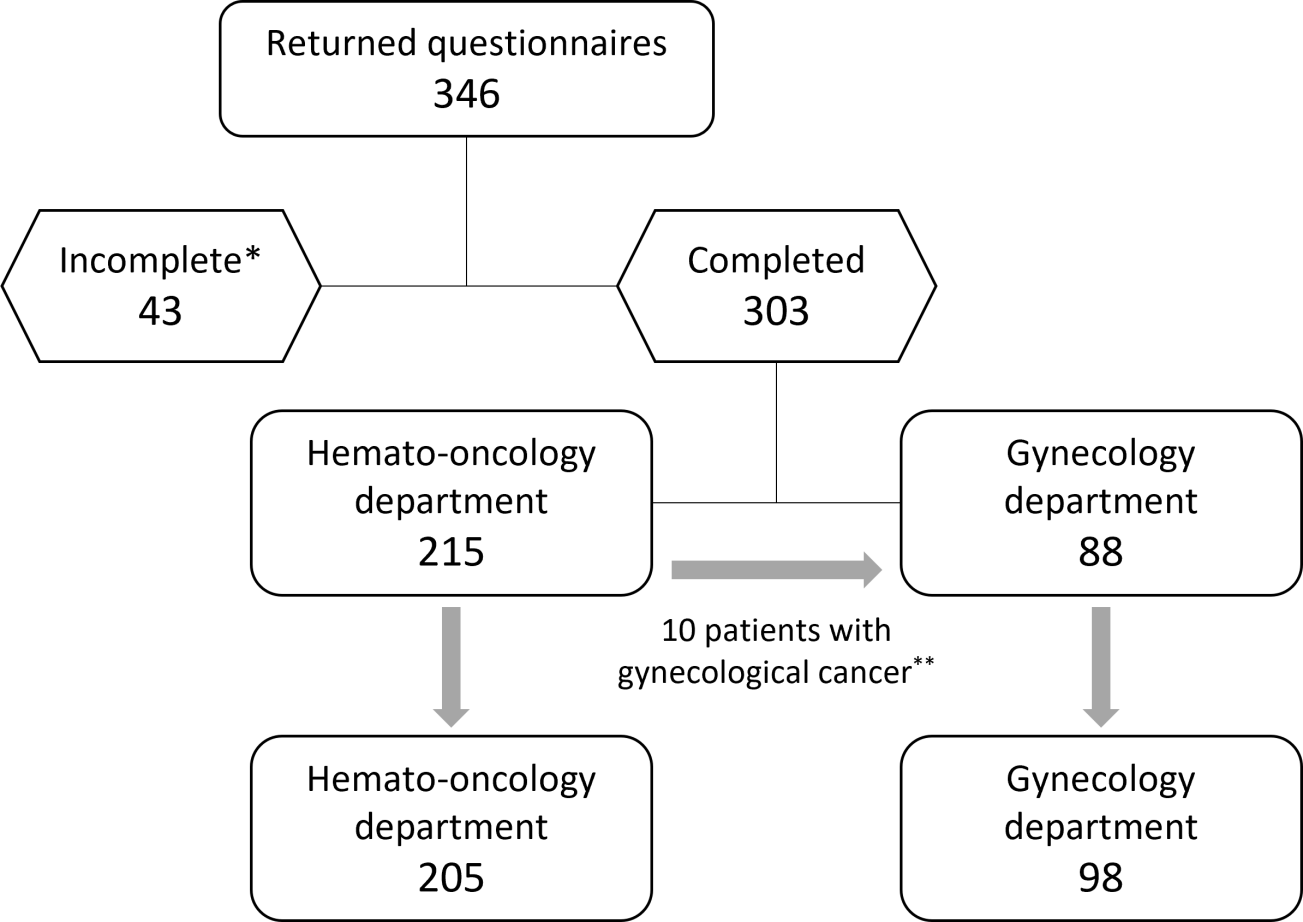
Study implementation. *Questionnaires with 2 or fewer missing variables were defined as “complete.” **For the purpose of this analysis, 10 patients with gynecological cancer who were receiving care at the hemato-oncology were moved into the group derived from the gynecology department.

Of 403 eligible patients approached, 346 returned the questionnaire, yielding a response rate of approximately 85.9%. In total, 303 (87.6%) of them were categorized as complete. A total of 97.3% (285/293) were oncology patients. As a few patients with multiple sclerosis were receiving chemotherapy in the hemato-oncology department, our collective also included 2.7% (8/293) of patients without cancer. In total, 10 patients did not specify their diagnosis, and due to the anonymous nature of the questionnaire, it was not possible to obtain this information. Figure 1 provides an overview of the recruitment and participation process.

### Demographics

The mean age of the respondents was 59 (SD 14.064) years. In total, 55.8% (169/303) were women, and 44.2% (134/303) were men. Table 1 shows the distribution of the participants’ primary diagnoses. The most common primary diagnosis was a gynecological tumor, followed by gastrointestinal tumors (defined as a tumor on an organ located in abdominal cavity, with the exception of kidney).

**Table 1.** Participants’ gender and diagnoses.

	Values, n (%)
Gender (n=303)	
Women	169 (55.8)
Men	134 (44.2)
Diagnosis (n=294)	
Gastrointestinal tumor^a^	88 (29.9)
Gynecological tumor^b^	96 (32.7)
Lymphoma	25 (8.5)
Neuroendocrine tumor^c^ (undefined)	13 (4.4)
Sarcoma	9 (3.1)
Urological tumor^d^	17 (5.8)
Bronchial carcinoma	6 (2.0)
Leukemia	5 (1.7)
Oropharyngeal tumor	4 (1.4)
Others	31 (10.5)

a All tumors of the following organs were designated as gastrointestinal tumors: liver, gall bladder, bile ducts, stomach, intestine (small and large), pancreas, esophagus, and any neuroendocrine tumors located in these mentioned organs.

b A total of 60.4% (58/96) of these were mamma carcinoma; all participants with other gynecology-related cancers were counted to “others.”

c Other neuroendocrine tumor not located in abdominal cavity.

d Urological tumors we defined kidney, ureter, bladder, urethra, and prostate. No participants had penile and testicle cancer.

### Access to and Use of the Internet

Only 3 patients did not have an internet-enabled device in their household, while 88.4% (268/303) of the patients reported having 2 or more internet-enabled devices. The most commonly used device was the mobile phone (199/288, 69.1%). In contrast, just over a third of the households had and used a stationary computer (Table 2).

**Table 2.** Ownership of web-enabled devices (n=303).

Web-enabled device available in the household	Values, n (%)
Smartphone	279 (92.1)
Notebook	209 (69)
Tablet	169 (55.8)
PC	110 (36.3)

### Internet Use Patterns and Digital Confidence

Most of the patients reported using the internet several times a day (244/299, 81.6%), and 91.6% (274/299) reported using the internet at least once a day (Table 3). In total, 17 (5.6%) patients (average age 73, SD 9.5 years) reported technical difficulties and no perceived benefit as their reasons for hardly using the internet. Additionally, 4 patients declared themselves as noninternet users and were therefore excluded from the complete final dataset. In total, 299 of the 303 questionnaires were included in the final results for this part of the evaluation.

The frequency of internet use was age dependent and increased with decreasing age (ρ=0.211; *P*<.001). In total, 40% (119/298) of the internet users always felt confident navigating the internet. A total of 18.5% (55/298) reported feeling confident in their ability to use the internet most of the time, and 33.9% (101/298) reported feeling confident using simple apps. Only 7.7% (23/298) reported that they did not feel at all confident using the internet. In total, 70% (208/297) reported that they have installed an app, and 9.4% (28/297) estimated their ability for this purpose as sufficient. A total of 17.5% (52/297) required help from others when installing apps; yet, only 3% (9/297) were of the opinion that they do not have the skills required to install an app.

Among the internet users, the majority (254/293, 86.7%) could receive updates and install them and have the current software version installed (shown in Table 4). These data are also reflected by the age of the devices owned by most patients. More than half of the devices (201/299, 67.2%) were at most 3 years old.

**Table 3.** Internet use.

Frequency of internet use	Values (n=299), n (%)	Age (years), mean (SD)
Minimum 1 time per day	274 (91.6)	57.77 (14.082)
Minimum 1 time per week	18 (6)	64.39 (11.387)
Less often	7 (2.3)	65.29 (10.242)

**Table 4.** Characteristics of most used devices.

	Values, n (%)
Age of device (years) (n=299)	
<1	49 (16.4)
1‐3	152 (50.8)
3‐5	71 (23.7)
>5	27 (9)
Operating system^a^ (n=292)	
Android	110 (37.7)
iOS	98 (33.6)
Windows	89 (30.5)
Other	19 (6.5)
Current software version (n=293)	
Updates available	254 (86.7)
Updates available but not installed	24 (8.2)
No updates available	15 (5.1)

a The questionnaire items targeted the primary internet-enabled device used by each participant. Nonetheless, some participants gave multiple answers.

### Use of the Internet and eHealth Apps

In total, 72.6% (217/299) of the patients reported using the internet for medical-related queries. These patients most frequently searched for information regarding their medical condition (170/299, 56.9%), followed by communication with the health insurance (112/299, 37.5%). Women tended to use the internet more frequently compared to men to communicate with other survivors of cancer (φ=0.184; *P*=.001). More than a third of the internet users already used 2 or more of the eHealth apps (Table 5). Younger participants tended to use eHealth apps more often than older ones (ρ=0.295; *P*<.001). A correlation was also found between the number of used apps and the increasing perceived competence in navigating the internet (ρ=0.320; *P*<.001) and navigating apps (ρ=0.330; *P*<.001). Perceived confidence navigating the internet and apps was significantly associated with a principal readiness to use more eHealth offers in the future (ρ=0.373 and 0.490, respectively; *P*<.001). Table 5 illustrates that patients most frequently reported readiness to use web-based platforms for communication with health care professionals and their health insurance. The least popular apps among this patient group were eHealth apps for the purpose of prevention. In total, 87.3% (261/299) of the internet users did at least specify that they are prepared to use 1 or more of the listed eHealth apps. However, increasing age correlated negatively with the number of eHealth apps that the participants were willing to use in the future (ρ=0.390; *P*<.001). In fact, significantly younger patients reported readiness to digitally manage health data for the following purposes: communicating with health care professionals, health insurance companies, or platforms certified by the government (*P*≤.001). Similar gender-related patterns could be detected. Between both collectives, our data revealed that men rather than women prefer to manage their health data digitally (φ=0.141; *P*=.02).

**Table 5.** Use of eHealth.

	Values, n (%)
Patients reported using the following eHealth apps (n=299)	
Searching for information	170 (56.9)
Communication with health care providers	40 (13.4)
Communication with health insurance	112 (37.5)
Communication with other survivors^a^	23 (7.7)
Keeping a symptom diary	22 (7.4)
Tracking vital parameters	27 (9)
Therapy apps^b^	7 (2.3)
Readiness to use the following eHealth apps (n=297)	
Organization of health data	200 (67.3)
Communication with health care providers	235 (79.1)
Communication with health insurance^c^	222 (74.5)
Use of a medical app approved by the German government^d^	190 (67.4)
eHealth apps for the purpose of (n=299)	
Health care^e^	179 (59.9)
Administrative processes	193 (64.5)
Prevention	110 (36.8)
Research	161 (53.8)
Therapy programs	137 (45.8)

a Definition of cancer survivor: “One who remains alive and continues to function during and after overcoming a serious hardship or life-threatening disease. In cancer, a person is considered to be a survivor from the time of diagnosis until the end of life” [26].

b For example, Diabetes- or Tinnitus-Trainer.

c n=298.

d n=282.

e For example, a videoconference with a clinician.

The type of operating system also seemed to affect patients’ affinity toward eHealth apps. For the following analysis, the 3 most common operating systems were considered: Android, iOS, and Windows. Patients with iOS on their device more often reported the use of therapy apps (φ=0.125; *P*=.03)—including examples such as Diabetes-Trainer and Tinnitus-Trainer [27,28]—and, in comparison with Android users, were also tended to use the internet more often to search for medical information (φ=0.148; *P*=.04). Here, no association could be shown with age or gender.

### Digital Medicine and COVID-19

In total, 36% (107/298) of the patients had installed the German government–issued “Corona-Warn-App” (Corona-Tracing-App) on their mobile phones at the point of participation in this investigation. Patients who had installed the app were on average 55 (SD 14.327) years of age and were thereby significantly younger than those who had not yet installed the app (mean 60, SD 13.388 years; 2-tailed *t* test; *P*=.001). Among the 170 patients who did not yet have the app installed, 51.1% (87/170) reported that they did not use the “Corona-Warn-App” because they did not see a benefit. However, 27.6% (47/170) of patients reported that they did not use the “Corona-Warn-App” due to technical problems (incompatible operating system and problems with the installation), and 15.3% (26/170) of patients had not heard about the app. In total, 10% (17/170) of patients did not use the “Corona-Warn-App” because they did not want to share their infection status through a digital means. An additional 2.9% (5/170) of the patients did not want to know about their own infection status. A total of 24.7% (68/275) of participants reported that their internet use changed due to the pandemic. In fact, 5.8% (16/275) began to use eHealth apps during the pandemic. In total, 62.5% (10/16) of these patients had installed the government-issued “Corona-Warn-App” as the first eHealth app on their mobile phone. While decreasing age correlated with increased eHealth app use during the pandemic (ρ=0.367; *P*<.001), women tended to be more likely to increase their use during the pandemic, while men did not report any pandemic-related changes (Cramer φ=0.241; *P*=.001).

### Comparison Between the Collective of Gynecology and Hemato-Oncology

Patients from the gynecology and hemato-oncology departments were also analyzed separately and compared. Patients from the gynecology department were significantly younger than those of the hemato-oncology (56 years compared to 60 years; *P*=.01). When comparing the 2 collectives, patients from the gynecology department reported feeling more competent using the internet (*P*=.02), used the internet more frequently (*P*=.04), and also tended to use more eHealth apps (*P*=.004). Gynecology patients also reported using web-based forums more often compared to other patients (φ=0.183; *P*=.002). Furthermore, they tended to measure and report vital parameters more frequently within the apps (φ=0.139; *P*=.02). Additionally, patients from the gynecology department communicated with their health insurance through digital apps more frequently (φ=0.136; *P*=.02) and kept digital symptom diaries more often when compared to the patients from the hemato-oncology department (φ =0.140; *P*=.01).

## Discussion

### Principal Findings

The fact that the overwhelming majority of patients own and use an internet-enabled device provides the foundation for widespread use of eHealth apps. In fact, the majority of the patients reported feeling confident navigating the internet and apps most of the time. Among those who reported lower levels of internet literacy, almost all could receive help when required. It can thus be ascertained that the fundamental conditions, the technical resources, and the know-how for the implementation of digital health apps are all given. In fact, in comparison with other studies, access to and use of the internet is rather high for our collective [20,21,29]. This allows the assumption that implementing eHealth may be even simpler among our patients when compared to other patient settings.

Our patients reported using the internet most frequently to search for and obtain medical information. While these results are consistent with existing literature [18,19], our results do not show quite a high tendency to use the internet to seek information about their disease as previous literature reports. This could be due to the fact that patients from both the gynecology and hemato-oncology departments are routinely made aware of the problems of misinformation through the internet and explicitly instructed not to seek information about their disease through a Google search. Instead, they are provided with evidence-based websites they can refer to when necessary. The next most common reasons cited for internet use among our collective were as follows: communication with health insurance companies (112/299), communication with physicians (40/299), and tracking of vital parameters (27/299). The number of patients who communicate digitally with physicians among our collective is 6.2% higher than that which Makowsky et al [30] report among their collective. However, Makowsky et al [30] investigated internet use among healthy patients, and this may explain the discrepancy. Furthermore, 1 in 13 patients reported using web-based forums to interact with other survivors. These findings align with prior studies, suggesting that patients with cancer seek digital-based peer communication and emotional support, which may serve as a coping mechanism and source of empowerment [8,31,32]. This postulation may also be supported by the fact that women tended to be more likely to participate in such forums than men.

In congruence with previous results [9,21], age influences use as well as openness to eHealth. As expected, the younger the patient, the more frequent the internet use, and the more confident the patients are when navigating the internet. Generally, younger patients are more open to digital health apps. Nevertheless, studies show that the older generation is also becoming increasingly familiar with the internet [33], indicating that the target group for the use of eHealth will continue to grow. Our data reflect this trend by demonstrating that almost all of our patients are regular internet users even though the average age was 59 years.

Whereas a younger age is consistently associated with increased internet use and competency, data regarding gender differences are not as consistent. Our results show that women are more likely than men to use internet forums to communicate with other survivors, but men report a higher readiness for managing health data digitally. These results are consistent with Harrison et al [34], who also demonstrated that, on average, women seek social support more than men. Harrison et al [34] explained this difference in reference to the classical gender stereotype that women are more communicative than men. This may also explain why women are more likely to search the web for support or more ready to participate in support groups [31,32]. However, our data did not confirm gender as an overall predictor of eHealth app use. To explore this further and rule out gender bias, future studies should apply multivariate analyses that control for age and internet literacy.

Among the 3 most frequently used operating systems (Android, iOS, and Windows), our results showed that iOS users tend to be more open-minded toward eHealth apps in comparison to patients who use other operating systems. In fact, iOS users were significantly younger than patients who used other operating systems. However, we found no relationship between age or the current operating system with regard to the use of therapy apps and using the internet to seek information. This may suggest an existing relationship between the operating system and the readiness for using digital medicine. For example, Ubhi et al [35] demonstrated that iOS users more often installed an app to help them stop smoking.

In line with existing literature, our data show that internet literacy is a strong predictor of eHealth readiness, often moderated by sociodemographic factors such as age, gender, and education [26-28]. Therefore, we recommend that before the implementation of eHealth apps, it is important to be familiar with the potential patient’s internet literacy. However, if time is lacking, our results, as well as results from previous studies, indicate that studies with patients younger than the age of 65 years may be more successful [2,9,21].

Both internet use in general and the use of eHealth apps increased during the pandemic. This could be due to the fact that when faced with social distancing restrictions and fear of infection, the domain of eHealth and digital health experienced an upswing [36]. Algumzi [37] confirmed this postulation, reporting that during the pandemic, the use of eHealth apps such as telemedicine and digital health increased in acceptance and use. These authors suggest that the reason could be due to fear of infection. This is also reflected in the fact that during the pandemic, many health care facilities, including our clinical facilities, reduced in-person consultations and switched to telehealth or digital options [38,39]

Although studies with similar timelines report that between 46% and 66% of the population use the government-issued “Corona-Warn-App,” only 36% (107/298) of our collective installed the Corona-Warn-App. The main reason cited for not using the “Corona-Warn-App” among all the studies was that the patients did not perceive any benefit from the app. Whereas previous studies cite privacy concerns and the perception of being monitored by the government, our patients named these reasons only sporadically. Nevertheless, our patients were more likely to cite technical problems (47/170, 27.6%) as an obstacle compared to 3.5% reported by Horstmann et al [40]. Our results did reflect a previously reported relationship between decreasing age and use of the government-issued Corona-Warn-App [40,41].

Our data revealed an existing gap between reported readiness to use eHealth apps and the reality of current use. While 79.1% (235/297) of our population reported readiness to use digital apps to communicate with health care professionals, only 13.4% (40/299) reported that they currently use a digital app to communicate with health care professionals. Similarly, while 74.5% (222/298) of the patients reported readiness to use eHealth apps to communicate with health insurance companies, only half (112/222) were currently using such methods, although they are widely available.

Comparable studies reflect these results [19]. In fact, consistent evidence shows that although health care professionals see great potential for eHealth apps to augment care, the step to real use is still hindered by various practical factors such as data protection, time restraints, and resistance to change among health care professionals [42]. Beyond these structural and technical barriers, additional challenges must be considered—particularly concerns about data privacy, trust in digital tools, and the influence of physician encouragement, all of which play a critical role in patients’ willingness to engage with eHealth solutions [43].

To bridge this gap, several practical strategies could be implemented: physician-mediated introductions to digital tools during consultations may foster trust and promote engagement; intuitive, low-threshold app designs can reduce usability barriers [44,45]; and tailored support—such as one-on-one training or digital health navigators as introduced in a national study in Germany [46]—could help patients build both competence and confidence. System-level integration of eHealth into routine care processes may further normalize use and promote sustainable adoption.

As our participant pool was limited to patients receiving outpatient therapy in hemato-oncology and gynecology, further studies are needed to explore whether these observations hold true across other clinical settings.

### Practice Implications

The gap between readiness and actual use of eHealth apps among oncology patients is decreasing. A slow and careful introduction of eHealth apps into the clinical routine and understanding of the population increases the likelihood of success. In general, this population seems open to eHealth tools; however, dedicated resources for support and assistance may promote growth in real-time use.

### Limitations

This study has several limitations. First, convenience sampling was used to recruit patients, which may have led to participation only by those patients who were interested and open to the topic of digital health. To rule out skewed results, patients who did not participate were screened to rule out a lack of knowledge or use of the internet. Furthermore, patients who were not interested or did not use the internet were not excluded from completing the questionnaire. However, a small number of patients (n=4) reported that they did not use the internet. The significantly younger age of the gynecology patients [47,48] must be considered as a possible confounding factor when analyzing our results. However, although there was a mean age difference between the 2 groups, we did not find a relationship with age or gender difference in the willingness to use digital apps to keep symptom diaries in the entire collective. Therefore, it can be postulated that we accounted for this confounding factor adequately. Second, due to the cross-sectional design of this study, no causal inferences can be made regarding the relationship between age, digital literacy, and actual or intended eHealth use. Future longitudinal research is needed to better understand the temporal dynamics and potential causality of these associations. Finally, more demographic data—such as education level, socioeconomic status, or digital access barriers—could have allowed us to account for the influence of education level on patients’ responses, and this factor could be considered a limitation when interpreting our results.

### Conclusions

Our study demonstrates the existence of the necessary basic conditions for implementing the use of eHealth apps among our collective. In fact, the majority of our patients already use the internet regularly and reported a high readiness to use eHealth. Nevertheless, as only a small percentage of the population currently uses a digital app, although they have access to and are aware of their existence, this study identified a gap between reported readiness to use eHealth apps and the reality of current use. More comprehensive research is necessary in order to identify ways to close this gap. Nevertheless, reported acceptance of different apps is high in almost all domains of eHealth. Positive predictors for use of the internet and eHealth include declining age, higher internet literacy, current use of the iOS operating system, and the number of currently used eHealth apps. While men are more likely to use eHealth for organizational functions, women tended to search for emotional support. As the pandemic caused a surge in eHealth use and acceptance, this may be an impulse for further use of eHealth apps and create an ideal environment for conducting further studies on this topic.

## Supplementary material

10.2196/72614 Multimedia Appendix 1 Questionnaire.

## Abbreviations

AGO: Arbeitsgemeinschaft Gynäkologische Onkologie
DKG: German Cancer Society
SPCC: Sharing Progress in Cancer Care
WSG: Westdeutsche Studiengruppe
