# Supplementary material for: Health-Related Internet Use Among Outpatients Undergoing Cancer Treatment During the COVID-19 Pandemic: Cross-Sectional Survey Study
Source: JMIR Hum Factors. 2025 Aug 14;12:e72614. doi: 10.2196/72614 (PMC12352702; doi:10.2196/72614)
Supplement: Multimedia Appendix 1 [file humanfactors-v12-e72614-s001.pdf]

This survey was administered in German, and the questions were translated with equivalent meaning for this publication

Patient survey on Internet usage

Dear Patient,

the Cancer Center – CCC Munich LMU – is conducting a patient survey on the use of the Internet in connection with health topics. We kindly ask you to answer the following questions about your Internet usage.

Your answers will help us adapt the deployment of new digital possibilities to best meet your needs.

Thank you very much for your cooperation!

Please complete the questionnaire following the instructions carefully and answer the questions in order.

Firstly, a few questions about yourself, which we will need for statistical evaluation:

1. **What gender do you identify as?**
   - ☐ male
   - ☐ female

2. **Which year were you born?**

   ____

The remaining questions now concern your personal habits regarding Internet use:

3. **Does your household have one or more of the following electronic devices with Internet access? (Multiple answers are possible)**
   - ☐ Mobile phone/Cell phone
   - ☐ Tablet
   - ☐ Laptop
   - ☐ Desktop computer

   If you have selected more than one: Which device do you use most frequently? (Please choose only one answer)
   - ☐ Mobile phone/Cell phone
   - ☐ Tablet
   - ☐ Laptop
   - ☐ Desktop computer

If you do not use any internet-based device at all, please answer the following question. You do not need to continue filling out the questionnaire afterwards: For what reasons do you not use the Internet? (Multiple answers possible)

- ☐ High costs
- ☐ Too much effort
- ☐ No additional purpose
- ☐ Technical difficulties
- ☐ Concerns about privacy protection
- ☐ Visual or auditive problems
- ☐ Impairment of hand/fingers
- ☐ Other: ____

4. **How often do you access the Internet on your electronic device?**
   - ☐ Several times a day
   - ☐ Once a day
   - ☐ 3-5 x per week
   - ☐ 1-2 x per week
   - ☐ Min. 1-4 x per month
   - ☐ Less
   - ☐ Never

5. **How confident do you feel in navigating and using the Internet?**
   - ☐ I can't manage it at all.
   - ☐ I can use simple applications, download apps, and navigate.
   - ☐ I know a few tricks and feel confident most of the time when using internet-based applications.
   - ☐ I always manage and can easily navigate internet-based applications.

6. **Are you familiar with installing apps on your internet-enabled device?**
   - ☐ Yes, I already installed an app
   - ☐ I haven't yet installed an app myself, but I judge my Internet skills to be sufficient to manage it.
   - ☐ I have not yet installed an app myself, but I could get assistance from family or friends to do so.
   - ☐ No, I don't feel able to install an app myself and I also have no way to get assistance with it.

7. **How old is your most frequently used internet-enabled device?**
   - ☐ < 1 year
   - ☐ 1 – 3 years
   - ☐ 3 – 5 years
   - ☐ > 5 years

8. **Which operating system does your most frequently used internet-enabled mobile device run on?**
   - ☐ Android
   - ☐ IOS (Apple)
   - ☐ Windows
   - ☐ Symbian OS
   - ☐ BlackBerry OS
   - ☐ Firefox OS
   - ☐ MacOS
   - ☐ Linux

9. **Is your internet-enabled device running the current software version from its provider?**
   - ☐ Yes, I regularly receive updates and install them.
   - ☐ No, I receive updates but do not install them.
   - ☐ No, my device no longer receives updates due to its age.

10. **Have you already used one or more of the aforementioned internet-based health applications (e-health applications) on your internet-enabled device?**
    - ☐ Searching for information on health topics
    - ☐ Communication with the treatment team
    - ☐ Communication with the health insurance provider
    - ☐ Forums for exchanging with other affected individuals
    - ☐ Keeping illness diaries
    - ☐ Measuring vital parameters (e.g., blood pressure, weight, ECG)
    - ☐ Therapy-APPs (eg., Diabetes-Trainer, Tinitus-Trainer)

11. **Would you be willing to use a data-secure internet-based platform to manage or record health data?**
    - ☐ yes
    - ☐ no

12. **Would you be willing to use a data-secure internet-based platform to communicate with your treatment team?**
    - ☐ yes
    - ☐ no

13. **Would you be willing to use a data-secure internet-based platform to communicate with your health insurance provider?**
    - ☐ yes
    - ☐ no

14. **Would you be willing to use a data-secure internet-based platform for the following purposes?**
    - ☐ Healthcare services (e.g., video consultation with a doctor)
    - ☐ Administrative processes (e.g., scheduling appointments online, submitting prescriptions online)
    - ☐ Prevention (e.g., online course for cancer prevention)
    - ☐ Research (e.g., participation in studies with online questionnaires)
    - ☐ Therapy programs (e.g., online seminars or training therapy for cancer patients)

15. Would you use a data-secure internet-based platform that has been reviewed and approved by the Federal Institute for Drugs and Medical Devices (BfArM)?

☐ yes ☐ no

16. Are you using the Corona-Warn-App on your smartphone/mobile phone?

☐ yes ☐ no

If you answered the question with no: For what reason do you not use the Corona-Warn-App?

☐ I don't know this app
☐ The app does not run on my phone's operating system.
☐ I don't know how to install this app
☐ I do not want to know my infection risk with the coronavirus.
☐ I think i do not get any advantage by using this app
☐ I do not want to share my infection status with the public (not even anonymously).

Other: ____
